# Influence of synchronous primary care telemedicine versus in-person visits on diabetes, hypertension, and hyperlipidemia outcomes: a systematic review

**DOI:** 10.1186/s12875-022-01662-6

**Published:** 2022-03-21

**Authors:** Russyan Mark S. Mabeza, Kahtrel Maynard, Derjung M. Tarn

**Affiliations:** 1 David Geffen School of Medicine at UCLA, University of California Los Angeles, 10833 Le Conte Avenue, Los Angeles, CA 90095 USA; 2 Brown University, 69 Brown Street, Providence, RI 02912 USA; 3 Department of Family Medicine, David Geffen School of Medicine at UCLA, University of California Los Angeles, 10833 Le Conte Avenue, Los Angeles, CA 90095 USA

**Keywords:** Telemedicine, Primary care, Diabetes, Hypertension, Hyperlipidemia

## Abstract

**Background:**

Telemedicine can be used to manage various health conditions, but there is a need to investigate its effectiveness for chronic disease management in the primary care setting. This study compares the effect of synchronous telemedicine versus in-person primary care visits on patient clinical outcomes.

**Methods:**

A systematic review of studies published in PubMed and Web of Science between 1996 and January 2021 was performed using keywords related to telemedicine, diabetes, hypertension, and hyperlipidemia. Included studies compared synchronous telemedicine versus in-person visits with a primary care clinician, and examined outcomes of hemoglobin A1c (HbA1c), blood pressure, and/or lipid levels.

**Results:**

Of 1724 citations screened, 7 publications met our inclusion criteria. Included studies were published between 2000 and 2018. Three studies were conducted in the United States, 2 in Spain, 1 in Sweden, and 1 in the United Kingdom. The telemedicine interventions investigated were multifaceted. All included synchronous visits with a primary care provider through videoconferencing and/or telephone, combined with other components such as asynchronous patient data transmission. Five studies reported on HbA1c changes, 5 on blood pressure changes, and 3 on changes in lipid levels. Compared to usual care with in-person visits, telemedicine was associated with greater reductions in HbA1c at 6 months and similar HbA1c outcomes at 12 months. Telemedicine conferred no significant differences in blood pressure and lipid levels compared to in-person clinic visits.

**Conclusions:**

A systematic review of the literature found few studies comparing clinical outcomes resulting from synchronous telemedicine versus in-person office visits, but the existing literature showed that in the primary care setting, telemedicine was not inferior to in-person visits for the management of diabetes, hypertension, or hypercholesterolemia. These results hold promise for continued use of telemedicine for chronic disease management.

**Supplementary Information:**

The online version contains supplementary material available at 10.1186/s12875-022-01662-6.

## Background

Telemedicine is defined as the use of telecommunication and information technologies to provide clinical health care at a distance [1–3]. It includes a diverse array of technologies, such as synchronous virtual visits through videoconferencing or telephone consultations, and asynchronous transmission and interpretation of clinical data (e.g., blood pressure readings, daily weights) [1, 4].

Due to the COVID-19 pandemic, telemedicine use increased dramatically [5], with a 154% increase in telemedicine encounters during the last week of March 2020 compared to the same surveillance period in 2019 [6]. Virtual visits reduced concerns regarding disease transmission and preserved personal protective equipment during the pandemic. Yet they also benefited patients with limited mobility [7] and difficulties with transportation or with taking time off work for appointments [8, 9]. In the United States, temporary federal and state regulatory changes during the pandemic allowed for greater patient access to telemedicine [5]. Evidence of the effectiveness of these telemedicine encounters on clinical outcomes would support the utility of continued use of telemedicine.

Studies have shown that telemedicine results in good clinical outcomes across various healthcare settings, including psychiatry [10, 11], ophthalmology [12, 13], post-surgical rehabilitation [14, 15], and malnutrition management [16]. Telemedicine may also be particularly beneficial for chronic disease management, but there is a need to understand the effect of telemedicine encounters in the primary care setting on clinical outcomes for patients with chronic diseases. Telemedicine interventions for chronic disease management have mostly investigated team-based care with intensive counseling, many using remote monitoring devices [17–21]. Systematic reviews are lacking on the influence of synchronous telemedicine encounters with a primary care provider on clinical outcomes. The objective of this study is to perform a systematic review of literature to examine the effect of synchronous telemedicine versus in-person primary care visits on clinical outcomes in patients with diabetes, hypertension, and hyperlipidemia.

## Methods

### Literature search strategy

PubMed and Web of Science were electronically searched to find relevant studies published between 1996 and January 19, 2021. We searched PubMed for Medical Subject Headings (MeSH terms) and key words in titles and abstracts. We searched Web of Science for Web of Science Keywords Plus function terms. Searches included terms related to telemedicine, telehealth, telecare, virtual visit, videoconferencing, primary care, diabetes, hypertension, and hyperlipidemia. Reviews, perspectives, commentaries, and case reports were excluded in the initial search. The complete search strategy can be seen in Additional file 1.

### Study selection

We combined PubMed and Web of Science searchers and removed duplicate articles. Two independent reviewers (RMM and KM) screened and assessed the titles and abstracts that were captured in the initial search for relevance, and selected studies for further review. Non-English manuscripts were excluded from full-text review. The criteria for inclusion are outlined in Table 1. Studies selected for full-text review were those that compared synchronous telemedicine encounters to in-person office visits, occurred in the primary care setting, and reported clinical outcomes related to diabetes, hypertension, or hyperlipidemia. Studies were excluded if virtual visits were not conducted in the primary care setting, there was no synchronous interactive component between the patient and provider, or if the telemedicine intervention was not provided by a primary care provider. Primary care providers included family medicine and internal medicine physicians and nurse practitioners, while excluded healthcare professionals were nurses, pharmacists, dietitians, endocrinologists, and case managers. Studies that focused on pediatric and obstetric populations were excluded. Results from the two reviewers were compared, and differences in their assessment of 6 studies were resolved by consensus and input from a third reviewer (DMT). These studies were ultimately excluded from the analysis because they did not meet complete inclusion criteria.

**Table 1:** Description of the PICOS criteria used in the present systematic review

Criteria	Description
Participants	Non-pregnant persons aged 18 years and above with diabetes, hypertension, or hyperlipidemia
Intervention	Synchronous telemedicine encounters provided by a primary care provider (family/internal medicine physicians and nurse practitioners)
Comparison	In-person primary care visits
Outcomes	*Primary outcome measures:* Changes in HbA1c, blood pressure (systolic/diastolic), and total cholesterol levels. *Secondary outcome measures:* Changes in LDL-C and triglyceride levels.
Study design	Randomized controlled trials, non-randomized controlled trials, retrospective, prospective, and matched cohort studies

### Data extraction

For each study, we extracted information regarding healthcare setting, country, study design, control group characteristics, and sample size. Additionally, we searched each study for patient age and sex composition, provider characteristics, patient inclusion criteria, and clinical outcomes assessed. Telemedicine interventions were examined for mode of contact (e.g., by videoconferencing or telephone) and option for asynchronous communication between the patient and primary care provider. The primary outcomes of interest were changes in hemoglobin A1c (HbA1c), systolic and diastolic blood pressures, and total cholesterol levels. Secondary endpoints included low-density lipoprotein-cholesterol (LDL-C) and triglyceride levels. Outcome measures were collated over the intervention period along with the timeframe of assessment.

## Results

### Study characteristics

**Fig. 1:**
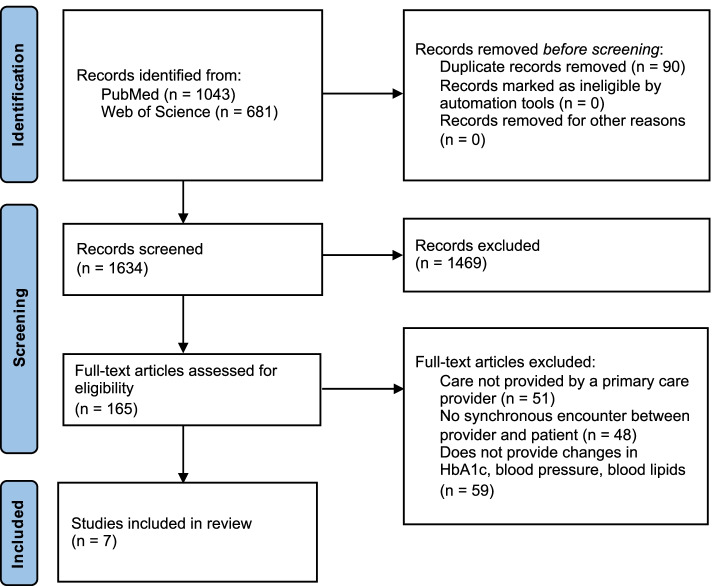
PRISMA flow diagram

Our search yielded 1043 articles from PubMed and 681 from Web of Science (Fig. 1). After removal of duplicate articles and abstract screening, 165 full-text articles were reviewed, of which 7 met criteria for inclusion in this review [22–28]. Table 2 summarizes major study characteristics using the PICO designations described in Table 1. Relatively few studies were identified in the 1990s; the majority of the manuscripts were published between 2000 and 2018. Four were conducted in Europe [23–26] and 3 in the United States [22, 27, 28]. Four studies were conducted across multiple health care clinics or facilities [23, 24, 26, 28] while 3 were single-center studies [22, 25, 28]. Four studies were conducted in urban settings [22, 24, 26, 28], 2 in rural [ 23, 27], and one across multiple cities with varied access to physicians [25]. Six studies were prospective [22–27] and all but one were randomized [27]. One study was a retrospective cohort study [28]. Sample sizes ranged from 28 to 1786 patients. Two studies investigated diabetes [22, 25], 2 studied hypertension [23, 28], and 3 examined diabetes, hypertension, and hyperlipidemia [24, 26, 27].

**Table 2:** Major study characteristics

Study	Healthcare Setting	Country	Study Design	Control Group Conditions	Patient Sample Size	Patient Age (Years)	Patient Sex (% Female)	Provider Characteristics	Patient Inclusion Criteria	Clinical Outcomes Assessed
Whitlock et al. (2000) [22]	Single primary care clinic	United States	RCT	Usual care	28	Study: 61.5 (41–73)^a^ Control: 59 (32–75)^a^	Study: 60 Control: 62	Primary care physician	HbA1c > 8.0%	HbA1c
Nilsson et al. (2009) [23]	Health centers	Sweden	Matched cohort study	Usual care with physician at a separate but similar healthcare center	273	Study: 65 (57–63)^a^ Control: 65 (57–63)^a^	Study: 54 Control: 54	Primary care physician	SBP > 140 DBP > 90	Blood pressure
Rodriguez-Idígoras et al. (2009) [24]	Family medicine practices	Spain	RCT	Usual care	328	Study: 63.3 (61.6–65.0)^b^ Control: 64.5 (63.0–66.1)^b^	Study: 46 Control: 51	Family physician	Age > 30 years Type 2 diabetes Self-monitoring for diabetes	HbA1c, blood pressure, total cholesterol, LDL-C
Esmatjes et al. (2014) [25]	Single outpatient clinic	Spain	RCT	5 face-to-face appointments with physician	154	Study: 32.2 ± 10.1^c^ Control: 31.5 ± 9.0^c^	Study: 57.6 Control: 52.6	Primary care provider	Aged 18–55 years Type 1 diabetes ≥ 5 year duration HbA1c > 8.0% Using multiple insulin doses per day Testing blood glucose at home at least 3 times/day Has Internet access at home	HbA1c
Basudev et al. (2015) [26]	General practices	United Kingdom	Prospective study	Usual care	208	Study: 60.5 ± 12.3^c^ Control: 59.3 ± 12.0^c^	Study: 45.2 Control: 40.4	Primary care and specialist diabetes teams	Age ≥ 18 years old Type 2 diabetes > 1 year duration HbA1c > 8.5%	HbA1c, blood pressure, total cholesterol
Tokuda et al. (2016) [27]	Single community-based outpatient clinic	United States	Prospective study	Regular individual visits with primary care physician every 4–6 months	100	Study: 60.4 ± 1.4^d^ Control: 61.6 ± 1.1^d^	Study: 0 Control: 10.1	Nurse practitioner and clinical pharmacist	HbA1c ≥ 7%	HbA1c, blood pressure, LDL-C, triglycerides
Levine et al. (2018) [28]	Primary care clinics	United States	Retrospective cohort study	Usual care	1786	Study: 61 (60–61)^b^ Control: 60 (59–61)^b^	Study: 44 Control: 42	Primary care physician	Engagement in a virtual visit for hypertension (intervention group) or principal diagnosis of essential hypertension (control group)	Blood pressure

Age is reported as median with interquartile range,^a^ mean with 95% confidence interval,^b^ mean with standard deviation,^c^ or mean with standard error ^d^

### Participant characteristics

All studies included adults at least 18 years of age. Eligibility criteria for studies focusing on diabetes required patients to have HbA1c levels of at least 7% [27], 8.0% [22, 25], and 8.5% [26]. Two required patients to be self-monitoring for diabetes [24, 25]. One study examining blood pressure as the outcome measure required participants to have blood pressures greater than 140/90 [23], while another study on blood pressure required an ICD-9 diagnosis of hypertension [28]. Only 2 studies provided information on the number of primary care providers involved in the study, with one reporting involvement of 2 providers [22] and the other 35 [24].

### Intervention characteristics

Multiple modalities were described under the umbrella of telemedicine, including face-to-face videoconferencing, telephone consultations, and self-monitoring devices, but all examined interventions included a synchronous encounter with a primary care provider. Table 3 summarizes the intervention components utilized by each study.

**Table 3:** Study intervention components

Intervention components	Whitlock et al. (2000) [22]	Nilsson et al. (2009) [23]	Rodriguez-Idígoras et al. (2009) [24]	Esmatjes et al. (2014) [25]	Basudev et al. (2015) [26]	Tokuda et al. (2016) [27]	Levine et al. (2018) [28]
Videoconferencing	✓	✓		✓	✓	✓	✓
Telephone visit	✓		✓			✓	✓
Asynchronous messaging			✓	✓			✓
Remote self-monitoring devices	✓		✓	✓		✓	✓
Real-time transmission of patient data			✓	✓			

Six of the 7 studies used a videoconferencing system (involving a computer-based video communication platform) to conduct telemedicine encounters [22, 23, 25–28]. One utilized a tele-assistance system involving patient and physician mobile phones [24]. Three studies utilized only video [23, 25, 26], 3 used both video and telephone [22, 27, 28], and 1 used only telephone encounters [24]. All studies originating in the United States employed both videoconferencing and telephone encounters [22, 27, 28], while those from other countries included only videoconferencing [23, 25, 26] or telephone consultations [24]. Only the two studies from Spain used real-time transmission of patient data [24, 25].

In addition to the synchronous interventions, 1 study utilized a messaging system wherein patients could communicate with their provider via a text message in less than 500 words [25], while another 2 studies offered patients the ability to message providers through a patient portal [24, 28]. In one study, structured digital exchanges between patient and primary care provider prompted telemedicine visits. Patients in this study entered blood pressure readings, binary responses regarding medication adherence, free text responses regarding medication side effects, and questions for the ordering clinician [28].

Study interventions often included utilization of remote monitoring devices [22, 24, 25, 27, 28]. Three studies used remote monitoring to guide patient counseling and treatment [22, 24, 25]. Patients in 3 studies [22, 24, 25] were provided with glucometers for home self-monitoring while patients in 2 other studies [27, 28] self-monitored using their own blood pressure cuffs. One study required intervention group subjects to use a sphygmomanometer attached to the telemedicine device provided by the investigators [22].

Four studies included interactions with members of a multidisciplinary healthcare team [23, 24, 26, 27]. In one study, participants worked with diabetes-trained clinical pharmacists in addition to their primary care providers [27]. In another, patients received supplemental education from specialized diabetes nurses [24]. Another study coupled telemedicine visits with in-depth primary care-specialist provider meetings to review the care of participating patients [26].

### Diabetes outcomes

Table 4 summarizes the clinical outcomes assessed in each study. The 5 studies assessing diabetes outcomes examined HbA1c levels at 4 different time points, ranging from 3 to 12 months [22, 24–27]. Only 3 of the 5 studies compared the intervention and control groups for changes in HbA1c levels [24, 26, 27]. These studies showed that compared to usual care with in-person visits, telemedicine was associated with significantly greater HbA1c improvements at 5 and 6 months [24, 27] and similar HbA1c outcomes at 12 months [24, 26]. Two other studies reported significantly decreased HbA1c levels within the intervention and control groups but did not compare the intervention and control groups [22, 25].

**Table 4:** Changes in clinical outcomes assessed

Study		Diabetes				Hypertension			Hyperlipidemia	
		Change in HbA1c				Change in systolic/diastolic blood pressure			Change in total cholesterol/LDL-C/triglyceride	
		3 months	5 months	6 months	12 months	5 months	12 months	Not specified	5 months	12 months
Whitlock et al. (2000) [22]	Intervention	↓*	–	–	–	–	–	–	–	–
	Control	↓	–	–	–	–	–	–	–	–
Nilsson et al. (2009) [23]	Intervention	–	–	–	–	–	–	↓^b^ / ↓^b^	–	–
	Control	–	–	–	–	–	–	↓ / ↓	–	–
Rodriguez-Idígoras et al. (2009) [24]	Intervention	–	–	↓***^a^	↓*^b^	–	↓* / ↓*	–	–	↓*/↓*/—
	Control	–	–	↓*	↓	–	↓ / ↓	–	–	—/↓*/—
Esmatjes et al. (2014) [25]	Intervention	–	–	↓***	–	–	–	–	–	–
	Control	–	–	↓***	–	–	–	–	–	–
Basudev et al. (2015) [26]	Intervention	–	–	–	↓^b^	–	↓^b^ / ↓^b^	–	–	↓^b^/—/—
	Control	–	–	–	↓	–	↑ / ↓	–	–	↓/—/—
Tokuda et al. (2016) [27]	Intervention	–	↓^a^	–	–	↓*^b^/ ↓*^b^	–	–	—/↓^b^/↓^b^	–
	Control	–	↑	–	–	↓ / ↓	–	–	—/↓/↑	–
Levine et al. (2018) [28]	Intervention	–	–	–	–	–	–	↓/—	–	–
	Control	–	–	–	–	–	–	↓/—	–	–

*p < 0.05, **p < 0.01, ***p < 0.001 Study reported statistical significance between baseline and outcome in either the intervention or control group at the specified duration

^a^Compared to the control group, the intervention group had greater improvements, *p* ≤ 0.05

^b^Study reported no statistical difference between intervention and control groups at *p* = 0.05

### Hypertension outcomes

Three studies examined hypertension control at 5 or 12 months [24, 26, 27], while 2 did not specify the period for outcome assessment [23, 28]. Three studies compared outcomes among intervention and control groups [23, 26, 27]. In these studies, the systolic and diastolic blood pressures in the telemedicine intervention groups did not differ significantly from those of the control groups at the end of the measurement period.

### Hyperlipidemia outcomes

Of the 3 studies that examined hyperlipidemia [24, 26, 27] 2 assessed LDL-C [24, 27], 2 assessed total cholesterol [ 24, 26], and 1 assessed triglyceride levels [27] as the outcome. Of the 2 studies comparing intervention and control groups, 1 demonstrated no statistically significant differences in LDL-C and triglycerides at 5 months [27] while another found no significant differences in total cholesterol changes at 12 months [26].

## Discussion

In this systematic review of the literature, we found that synchronous telemedicine encounters resulted in either improved or non-inferior diabetes, hypertension, and hyperlipidemia control compared to in-person primary care office visits. None of the studies examined showed inferior outcomes in patients receiving telemedicine encounters at any time point assessed. These results suggest that telemedicine is a viable option for chronic disease management in the primary care setting.

This study adds to the literature by systematically reviewing the evidence supporting the use of synchronous telemedicine encounters in the primary care setting for chronic disease management. Previous systematic reviews and meta-analyses have investigated the effect of telemedicine on chronic disease management, but many of these included studies focused solely on wearable devices or remote monitoring [29, 30]. Other reviews have examined outcomes such as medication adherence and health equity but did not focus on clinical outcomes [31, 32]. To our knowledge, no prior systematic review has investigated the impact of synchronous telemedicine encounters on clinical outcomes of diabetes, hypertension, and hyperlipidemia.

All studies examined in this review included a synchronous provider-patient telemedicine encounter, but there was notable heterogeneity in the interventions used. Most of the interventions included components in addition to the synchronous telemedicine encounter. Some included remote self-monitoring devices and real-time transmission of patient data, while others included asynchronous patient messaging. While most studies compared the intervention group to usual care, a few scheduled regular in-person visits or mandated a certain number of visits for patients in the control group.

Our findings hold promise for increased use of telemedicine for chronic disease management in the primary care setting, but further work is needed to better compare ‘real world’ telemedicine encounters to in-person office visits. Some of the studies provided intervention group patients with remote self-monitoring devices that were not given to control group patients. It is unknown how much these telemedicine components contributed to intervention effects, and whether the interventions would have achieved the same clinical outcomes without these additional components. Knowledge is also needed about whether provision of visits by video or telephone differentially influences patient outcomes. Furthermore, more work assessing the role of interdisciplinary teams, including specialists, social workers, and other healthcare professionals, on delivering telemedicine interventions for chronic disease management is warranted.

This study has several limitations. There was a paucity of literature regarding synchronous telemedicine’s use for chronic disease management by primary care providers, and most studies included components other than synchronous video or telephone interventions. Furthermore, studies focusing on elderly or fragile patients were lacking. Details on videoconferencing software platforms and blood pressure measurement devices used in the studies were limited. Not all studies directly compared clinical outcomes among intervention and control groups. Data regarding HDL and HDL/LDL ratios were not reported in the reviewed studies. Information was generally lacking about the racial/ethnic composition of participants.

## Conclusions

A systematic review of the literature found few studies comparing clinical outcomes resulting from synchronous telemedicine encounters versus in-person office visits. However, existing literature revealed that in the primary care setting, telemedicine was not inferior to in-person visits for diabetes, hypertension, and hyperlipidemia control. These results hold promise for increased use of telemedicine for chronic disease management.

## Supplementary Information

**Additional file 1.**

## Data Availability

Data sharing is not applicable to this article as no datasets were generated or analyzed during the current study.

## Abbreviations

HbA1c: Hemoglobin A1c
LDL-C: Low-density lipoprotein-cholesterol
SBP: Systolic blood pressure
DBP: Diastolic blood pressure
RCT: Randomized controlled trial

## References

[1] Field MJ, Telemedicine: a guide to assessing telecommunications in health care (1996). Institute of Medicine (US) committee on evaluating clinical applications of telemedicine.

[2] What is Telehealth?. 2018. Available at: https://catalyst.nejm.org/doi/full/10.1056/CAT.18.0268. Accessed July 19, 2021.

[3] Chaet D, Clearfield R, Sabin JE, Skimming K (2017). Council on ethical and judicial affairs American Medical Association: ethical practice in Telehealth and telemedicine. J Gen Intern Med.

[4] Baker J, Stanley A (2018). Telemedicine technology: a review of services, equipment, and other aspects. Curr Allergy Asthma Rep.

[5] Baum A, Kaboli PJ, Schwartz MD (2021). Reduced in-person and increased Telehealth outpatient visits during the COVID-19 pandemic. Ann Intern Med.

[6] Koonin LM, Hoots B, Tsang CA (2020). Trends in the use of Telehealth during the emergence of the COVID-19 pandemic — United States, January-march 2020. MMWR Morb Mortal Wkly Rep.

[7] Musich S, Wang SH, Ruiz J, Hawkins K, Wicker E (2018). The impact of mobility limitations on health outcomes among older adults. Geriatr Nurs.

[8] Syed ST, Gerber BS, Sharp LK (2013). Traveling towards disease: transportation barriers to health care access. J Community Health.

[9] Gleason RP, Kneipp SM (2004). Employment-related constraints: determinants of primary health care access?. PPNP..

[10] Reilly RO, Bishop J, Maddox K, Hutchinson L, Fisman M, Takhar J (2007). Is telepsychiatry equivalent to face-to-face psychiatry? Results from a randomized controlled equivalence trial. Psychiatr Serv.

[11] Egede LE, Acierno R, Knapp RG (2015). Psychotherapy for depression in older veterans via telemedicine: a randomised, open-label, non-inferiority trial. Lancet Psychiatry.

[12] Shi L, Wu H, Dong J, Jiang K, Lu X, Shi J (2015). Telemedicine for detecting diabetic retinopathy: a systematic review and meta-analysis. Br J Ophthalmol.

[13] Kawaguchi A, Sharafeldin N, Sundaram A (2018). Tele-ophthalmology for age-related macular degeneration and diabetic retinopathy screening: a systematic review and Meta-analysis. Telemed J E Health.

[14] Jiang S, Xiang J, Gao X, Guo K, Liu B (2018). The comparison of telerehabilitation and face-to-face rehabilitation after total knee arthroplasty: a systematic review and meta-analysis. J Telemed Telecare.

[15] Van Egmond MA, van der Schaaf M, Vredeveld T (2018). Effectiveness of physiotherapy with telerehabilitation in surgical patients: a systematic review and meta-analysis. Physiotherapy..

[16] Marx W, Kelly JT, Crichton M (2018). Is telehealth effective in managing malnutrition in community-dwelling older adults? A systematic review and meta-analysis. Maturitas.

[17] Timpel P, Oswald S, Schwarz PEH, Harst L (2020). Mapping the evidence on the effectiveness of telemedicine interventions in diabetes, dyslipidemia, and hypertension: an umbrella review of systematic reviews and Meta-analyses. J Med Internet Res.

[18] Kitsiou S, Paré G, Jaana M (2013). Systematic reviews and meta-analyses of home telemonitoring interventions for patients with chronic diseases: a critical assessment of their methodological quality. J Med Internet Res.

[19] Faruque LI, Wiebe N, Ehteshami-Afshar A (2017). Alberta kidney disease network. Effect of telemedicine on glycated hemoglobin in diabetes: a systematic review and meta-analysis of randomized trials. CMAJ..

[20] So CF, Chung JW (2018). Telehealth for diabetes self-management in primary healthcare: a systematic review and meta-analysis. J Telemed Telecare.

[21] Akbari M, Lankarani KB, Naghibzadeh-Tahami A (2019). The effects of mobile health interventions on lipid profiles among patients with metabolic syndrome and related disorders: a systematic review and meta-analysis of randomized controlled trials. Diabetes Metab Syndr.

[22] Whitlock WL, Brown A, Moore K (2000). Telemedicine improved diabetic management. Mil Med.

[23] Nilsson M, Rasmark U, Nordgren H (2009). The physician at a distance: the use of videoconferencing in the treatment of patients with hypertension. J Telemed Telecare.

[24] Rodríguez-Idígoras M, Sepúlveda-Muñoz J, Sánchez-Garrido-Escudero R (2009). Telemedicine influence on the follow--up of type 2 diabetes patients. Diabetes Technol Ther.

[25] Esmatjes E, Jansà M, Roca D, Pérez-Ferre N (2014). The efficiency of telemedicine to optimize metabolic control in patients with type 1 diabetes mellitus: Telemed study. Diabetes Technol Ther.

[26] Basudev N, Crosby-Nwaobi R, Thomas S, Chamley M, Murrells T, Forbes A (2016). A prospective randomized controlled study of a virtual clinic integrating primary and specialist care for patients with type 2 diabetes mellitus. Diabet Med.

[27] Tokuda L, Lorenzo L, Theriault A (2016). The utilization of video-conference shared medical appointments in rural diabetes care. Int J Med Inform.

[28] Levine DM, Dixon RF, Linder JA (2018). Association of Structured Virtual Visits for hypertension follow-up in primary care with blood pressure control and use of clinical services. J Gen Intern Med.

[29] Kamei T, Kanamori T, Yamamoto Y, Edirippulige S. The use of wearable devices in chronic disease management to enhance adherence and improve telehealth outcomes: a systematic review and meta-analysis. J Telemed Telecare. 2020. Advance online publication. 10.1177/1357633X20937573. 10.1177/1357633X20937573 32819184

[30] Zhang W, Cheng B, Zhu W, Huang X, Shen C (2021). Effect of telemedicine on quality of care in patients with coexisting hypertension and diabetes: a systematic review and meta-analysis. Telemed J E Health..

[31] Bingham JM, Black M, Anderson EJ (2021). Impact of Telehealth interventions on medication adherence for patients with type 2 diabetes, hypertension, and/or dyslipidemia: a systematic review. Ann Pharmacother.

[32] Turnbull S, Cabral C, Hay A, Lucas PJ (2020). Health equity in the effectiveness of web-based health interventions for the self-Care of People with chronic health conditions: systematic review. J Med Internet Res.

