# Supplementary material for: Influence of synchronous primary care telemedicine versus in-person visits on diabetes, hypertension, and hyperlipidemia outcomes: a systematic review
Source: BMC Prim Care. 2022 Mar 21;23:52. doi: 10.1186/s12875-022-01662-6 (PMC8936383; doi:10.1186/s12875-022-01662-6)
Supplement: Supplementary file 1 — Additional file 1. [file 12875_2022_1662_MOESM1_ESM.docx]

**APPENDIX 1**

**PubMed Search Strategy:**

(((telemedicine[MeSH Terms]) OR (videoconferencing[MeSH Terms])) OR ((((((Telemedicine[Title/Abstract]) OR (telehealth[Title/Abstract])) OR (eHealth[Title/Abstract])) OR (telecare[Title/Abstract])) OR ("virtual visit"[Title/Abstract])) OR ("video consultation"[Title/Abstract]))) AND (((((primary care[MeSH Terms]) OR (diabetes mellitus[MeSH Terms])) OR (hypertension[MeSH Terms])) OR (hyperlipidemia[MeSH Terms])) OR (((("primary care"[Title/Abstract]) OR (diabet*[Title/Abstract])) OR (hypertens*[Title/Abstract])) OR (hyperlipidemia[Title/Abstract])))

Filters: Clinical Study, Clinical Trial, Clinical Trial Protocol, Clinical Trial, Phase I, Clinical Trial, Phase II, Clinical Trial, Phase III, Controlled Clinical Trial, Observational Study, Pragmatic Clinical Trial, Randomized Controlled Trial, Validation Study

**Web of Science Search Strategy:**

Search Query:

TOPIC: (Telemedicine) *OR* TOPIC: (Telehealth) *OR* TOPIC: (Telecare) *OR* TOPIC: (Virtual visit) *OR* TOPIC: (Video consultation) *OR* TOPIC: (eHealth) *OR* TOPIC: (Videoconferencing) *AND* TOPIC: (Primary care) *OR* TOPIC: (Diabetes) *OR* TOPIC: (Hypertension) *OR* TOPIC: (Hyperlipidemia)

Refined by: WEB OF SCIENCE CATEGORIES: (MEDICINE GENERAL INTERNAL OR MEDICINE RESEARCH EXPERIMENTAL) AND [excluding] DOCUMENT TYPES: (EDITORIAL MATERIAL OR CORRECTION OR REVIEW OR PROCEEDINGS PAPER OR BOOK CHAPTER OR MEETING ABSTRACT)

Indexes=SCI-EXPANDED, SSCI, A&HCI, CPCI-S, CPCI-SSH, BKCI-S, BKCI-SSH, ESCI, CCR-EXPANDED, IC Timespan=All years
